# Comparison of an anthropomorphic PRESAGE® dosimeter and radiochromic film with a commercial radiation treatment planning system for breast IMRT: a feasibility study

**DOI:** 10.1120/jacmp.v15i1.4531

**Published:** 2014-01-06

**Authors:** Khalid Iqbal, Kent A Gifford, Geoffrey Ibbott, Ryan L Grant, Saeed Ahmad Buzdar

**Affiliations:** ^1^ Department of Radiation Physics The University of Texas M D Anderson Cancer Center Houston TX USA; ^2^ Department of Physics The Islamia University Bahawalpur Pakistan; ^3^ Department of Radiation Oncology Shaukat Khanum Cancer Hospital & Research Center Lahore Pakistan

**Keywords:** 3D dosimetry, PRESAGE®, IMRT, QA, EBT2 GAFCHROMIC film

## Abstract

This work presents a comparison of an anthropomorphic PRESAGE® dosimeter and radiochromic film measurements with a commercial treatment planning system to determine the feasibility of PRESAGE® for 3D dosimetry in breast IMRT. An anthropomorphic PRESAGE® phantom was created in the shape of a breast phantom. A five‐field IMRT plan was generated with a commercially available treatment planning system and delivered to the PRESAGE® phantom. The anthropomorphic PRESAGE® was scanned with the Duke midsized optical CT scanner (DMOS‐RPC) and the OD distribution was converted to dose. Comparisons were performed between the dose distribution calculated with the Pinnacle^3^ treatment planning system, PRESAGE®, and EBT2 film measurements. DVHs, gamma maps, and line profiles were used to evaluate the agreement. Gamma map comparisons showed that Pinnacle^3^ agreed with PRESAGE® as greater than 95% of comparison points for the PTV passed a ±3%/±3mm criterion when the outer 8 mm of phantom data were discluded. Edge artifacts were observed in the optical CT reconstruction, from the surface to approximately 8 mm depth. These artifacts resulted in dose differences between Pinnacle^3^ and PRESAGE® of up to 5% between the surface and a depth of 8 mm and decreased with increasing depth in the phantom. Line profile comparisons between all three independent measurements yielded a maximum difference of 2% within the central 80% of the field width. For the breast IMRT plan studied, the Pinnacle^3^ calculations agreed with PRESAGE® measurements to within the ±3%/±3mm gamma criterion. This work demonstrates the feasibility of the PRESAGE® to be fashioned into anthropomorphic shape, and establishes the accuracy of Pinnacle^3^ for breast IMRT. Furthermore, these data have established the groundwork for future investigations into 3D dosimetry with more complex anthropomorphic phantoms.

PACS number: 87.53.Jw, 87.55.D‐, 87.55.dk

## I. INTRODUCTION

The need for accurate, practical three‐dimensional dosimetry has become a necessary part of the radiation delivery and treatment process. IMRT commissioning is often performed with 2D relative dose measurements using radiochromic or radiographic film, diode arrays or ion chambers. At present, measuring 3D dose distributions is a difficult and time‐consuming task. Gel dosimeters have been used for 3D dosimetry and many studies demonstrate feasibility. However, gel dosimeters can be difficult to manufacture and read out, and may exhibit sensitivity of the response to oxygen.^(1)^ Recently, PRESAGE® has been studied as a radiochromic three‐dimensional dosimeter. PRESAGE® is composed of polyurethane, radiochromic components (leuco dyes), and halogen‐containing free radical initiators. PRESAGE® has an optical attenuation coefficient that changes linearly with absorbed dose. The combination of PRESAGE® and an optical CT scanner has addressed this need to measure the dose in three dimensions.^2^, ^3^, ^4^, ^5^, ^6^, ^7^, ^8^, ^9^, ^10^, ^11^, ^12^, ^13^ There are three characteristics of PRESAGE® that make it suitable as a radiochromic‐based dosimeter. First, PRESAGE® can be fashioned into an optically clear 3D solid. Second, PRESAGE® polymerizes at a relatively low temperature which minimizes undesired thermal oxidation reactions that contribute to enhanced background of the dosimeter. Third, PRESAGE® is a tissue‐equivalent material.^14^, ^15^, ^16^, ^17^

Many studies have been performed on the PRESAGE® dosimeters that show acceptable agreement between measured and reference doses. Sakhalkar et al.^18^, ^19^, ^20^ demonstrated that the PRESAGE®/optical CT system has excellent precision, accuracy, reproducibility, and robustness for 3D dosimetry. Oldham et al.^(1)^ showed that dose profiles and isodoses between the PRESAGE®/optical CT system, Eclipse, and EBT2 film demonstrated excellent agreement at all points except within 3 mm of the outer edge of the dosimeter. Further, Oldham et al showed that the gamma map and dose calculation comparisons in the absence of inhomogeneities were within ±3%/±3mm for 96% of the comparison points. Sakhalkar et al.^(21)^ also showed that relative dose profile comparisons between Eclipse, EBT2, and PRESAGE® were within 4%. The gamma comparisons showed that the calculations and measurements were within the gamma criterion of ±4% and ±3mm for >94% of comparison points except those points near the edges.

Previous work has focused on the basic dosimetric characteristics of PRESAGE® and investigation of the feasibility of the PRESAGE®/optical CT system for 3D dosimetry. The latter investigations involved delivering simple dose distributions or IMRT distributions to dosimeters fabricated in regular cylindrical shapes. The present study evaluates the feasibility of a breastshaped anthropomorphic PRESAGE® dosimeter, and builds on this earlier work by applying the PRESAGE®/optical CT system to the verification of a five‐field complex IMRT delivery for breast IMRT. To our knowledge, this is the first study of an anthropomorphic breast PRESAGE® dosimeter. Independent measurements were also performed using EBT2 GAFCHROMIC film as a verification check on the PRESAGE® and Pinnacle^3^ distributions.

## II. MATERIALS AND METHODS

### A. PRESAGE® dosimeter and optical CT scanning

The PRESAGE® dosimeter (Heuris Pharma LLC, Skillman, NJ) was molded from a batch of pre‐mold mixture which was comprised of a solvent, leuco dye, and halogen‐based free radical initiator. The formulation of PRESAGE® used in this study had $Z_{\text{eff}}$ of 7.6 according to Heuris Pharma, a physical density of 1.07 g/cm^3^ reported by the Pinnacle^3^ treatment planning system, and CT number of 122. The PRESAGE® dosimeter was fashioned into a breast shape. The physical dimensions of the breast PRESAGE® dosimeter were 13.8 cm (length) by 12.4 cm (width) and 4.6 cm (height). The dosimeter was scanned with the Duke Midsized Optical Scanner dedicated for the RPC (DMOS‐RPC) (Duke University, Durham, NC) using 1° per step to produce 360 projection images.^(22)^ Transverse images were reconstructed by filtered back projection to a 1 mm voxel edge.

**Figure 1:**
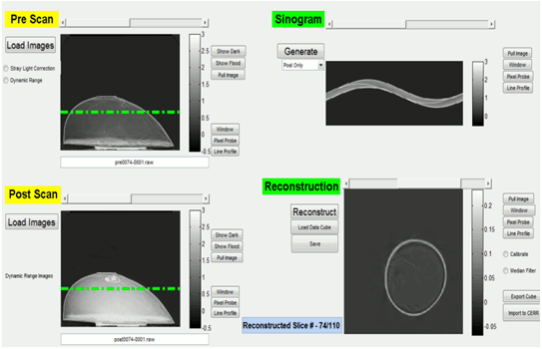
DMOS reconstruction graphical user interface: (upper left) projection images associated with the pre‐irradiation scan; (upper right) sonogram; (lower left) projection images of the post‐irradiation scan; (lower right) reconstructed transverse image. A correction for stray light is applied to each pre‐irradiation scan and post‐irradiation scan projection prior to reconstruction.

The DMOS‐RPC scanner (Fig. 1) consists of a matched telemetric source and image lens that provides a field of view (FOV) that is matched to the dimensions of the dosimeter. The DMOS‐RPC uses a near parallel diffused light source coupled to a matched telecentric lens and a CCD camera to image the PRESAGE®. The light passes through an aquarium with an antiglare coating which holds the PRESAGE® and matching fluid. The light is attenuated through the dosimeter and received by a telecentric lens. The matching fluid is composed of octyl salicylate, octyl cinnamate, and mineral oil in a combination that matches the index of refraction of the PRESAGE®. Edge artifacts arise from the reflection and refraction due to imperfect index of refraction matching between the mineral oil solution and the PRESAGE®.

### B. Treatment planning and delivery

**Figure 2:**
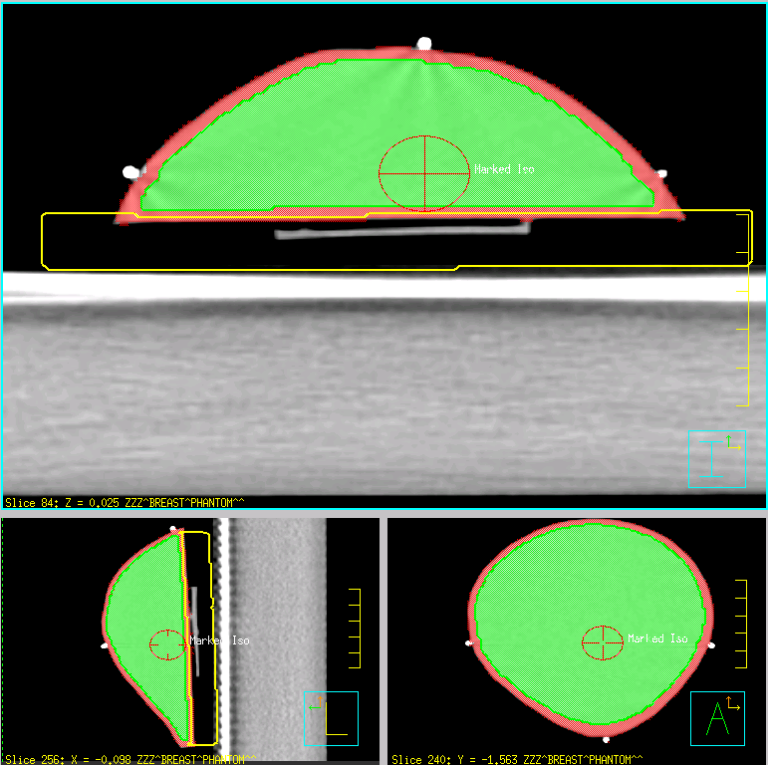
Axial, sagittal, and coronal views of the PRESAGE® breast. The regions of interest are breast (red), PTV (green), and lung avoidance (yellow).

A treatment planning X‐ray CT scan with a slice thickness of 1 mm was acquired of the breast PRESAGE® dosimeter using a GE CT scanner (GE Healthcare Technologies, Waukesha, WI). Subsequent to the CT scan, a pre‐irradiation optical CT was performed to assess any optical density changes from the CT acquisition. The CT data were exported to a Pinnacle^3^ v 9.0 treatment planning workstation (Royal Philips Electronics, Eindhoven, The Netherlands) where the treatment plan was created. The entire breast was contoured. The PTV was created by contracting the breast contour by 3 mm uniformly. A lung avoidance structure was created beneath the breast. These structures are illustrated in Fig. 2. PTV subvolumes were drawn inside the PTV to elucidate whether deeper regions of interest were affected less by edge artifacts. The PTV subvolumes were created by contracting the PTV by 1 mm, 3 mm and 5 mm isotropically. The IMRT plan was optimized to deliver 300 cGy to the PTV, while avoiding more than 225 cGy to the lung avoidance structure, with 5 gantry angles (265°, 300°, 0°, 60°, and 95°) and 6 MV. These five beams shared a common isocenter (the marked isocenter as shown in Fig. 2). Dose calculations were computed with the collapsed cone convolution algorithm with a 3 mm grid resolution.

The breast PRESAGE® was situated on a 5 cm thick block of solid water on the linac couch, and the lasers were aligned with the three BBs, as shown in Fig. 2. 300 cGy was delivered to the dosimeter with a 6 MV beam on a Varian 21EX linear accelerator (Varian Medical Systems, Palo Alto, CA). 300 cGy was chosen as to best maximize signal‐to‐noise ratio for the optical CT scanning for this batch of PRESAGE®.

Cuvettes with a volume of 1cm×1cm×3cm were irradiated in high‐impact polystyrene with a 6 MV beam on a Varian 21EX linear accelerator. Dose levels were 0, 300, 600, and 900 cGy. The linearity of the PRESAGE® dosimeter over this dose range has been demonstrated previously.^14^, ^15^, ^23^, ^24^, ^25^ The absorption of the material was determined by a Genesys 20 spectrophotometer (Thermo Fisher Scientific, Houston, TX) prior to and post irradiation and the OD was compared to the dose delivered to calculate the PRESAGE® calibration.

### C. Independent EBT2 film measurement

**Figure 3:**
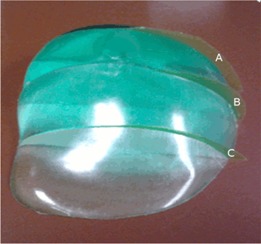
The PRESAGE® was cut into three axial sections. EBT2 film was inserted at the cut plane to provide an independent 2D measurement of the dose distributions in this plane.

An independent dose distribution verification was performed using GAFCHROMIC EBT2 film (ISP Corp, Wayne, NJ, USA). Temporal stability, directional independence and convenience of the self‐developing radiochromic film were the basic reasons to use EBT2 film.^(26)^ A PRESAGE® dosimeter was cut at three levels corresponding approximately to parallel axial planes, and pieces of EBT2 film were placed between the PRESAGE® sections, as shown in Fig. 3. The dosimeter with films inserted was irradiated with the original five‐field IMRT plan. An OD to dose curve was measured in solid water with 6 MV photons from a Varian 21EX. The films were digitized using a 48‐bit transmission/reflection flatbed photo‐scanner Epson‐10000XL (Epson America, Inc. Long Beach, CA). Each film was scanned in transmission mode and only the red channel was extracted for analysis because EBT2 film has a maximum response to red light at 633 nm.^(11)^ The calibration curve was applied to the EBT2 film to convert OD to dose.

### D. Data registration and dose analysis

The transverse images with dose distribution from DMOS‐RPC and the Pinnacle^3^ treatment plan were exported to Computational Environment for Radiotherapy Research program CERR (Memorial Sloan Kettering Cancer Center, New York, NY). The calculated dose distribution from Pinnacle^3^ was compared to the measured distributions from PRESAGE® and EBT2. EBT2 scans were analyzed using Image J software (National Institutes of Health, USA). At the time of this investigation, the CERR software did not have a true 3D gamma calculation, so all quantitative analysis between datasets was restricted to a slice‐by‐slice analysis using line profiles, DVHs, and 2D gamma maps.^(3)^ The gamma analysis criterion selected for the gamma map comparisons was ±3%/±3mm. Comparisons with EBT2 film were used as a second independent measurement to verify the accuracy of PRESAGE® and Pinnacle^3^.

## III. RESULTS & DISCUSSION

### A. PRESAGE® and EBT2 film calibration

**Figure 4:**
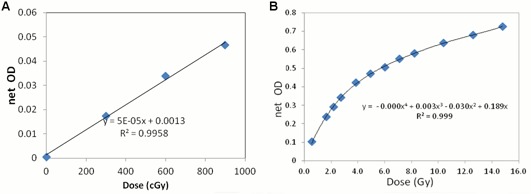
OD to dose curve for PRESAGE® (a); OD to dose curve for EBT2 film (b).

It was determined that the treatment planning CT scan of the breast PRESAGE® dosimeter did not produce any change in OD of the dosimeter. The radiochromic response was linear with a sensitivity of 0.0059 OD change for a 1 mm path length, as shown in Fig. 4(a). Figure 4(b) shows the EBT2 film OD to dose curve. The coefficients of variation for the PRESAGE® and EBT2 film were 1.0% and 0.75%, respectively.

### B. Dose‐volume histograms of the PTV

**Figure 5:**
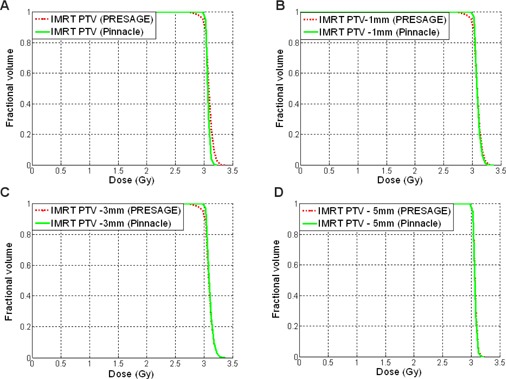
Dose‐volume histogram comparisons between the PRESAGE® and Pinnacle^3^: (a) PTV; (b) PTV 1 mm; (c) PTV

Figure 5 illustrates the PTV DVH comparisons of the five‐field breast IMRT plan between PRESAGE® and Pinnacle^3^. There are regions near the edges where the PRESAGE® measurements differ from Pinnacle^3^. This is due to edge artifacts, as observed in similar studies.^(19)^ Ninety‐five and one‐half percent (95.5%) of comparison points passed the ±3%/±3mm test criterion between Pinnacle^3^ and PRESAGE® for the PTV when the outer 8 mm of phantom were discluded.

The PRESAGE®‐determined PTV DVHs were slightly less homogenous than those calculated by Pinnacle^3^, with small regions of relative over‐ and underdose occurring near the edge of the breast phantom. For the PTV DVH, a maximum 5% dose difference was observed at 5% and 95% of the fractional volume, as shown in Fig. 5(a). It is likely that part of this difference is real and part is due to artifacts in the PRESAGE® distribution. The PTV subvolume DVHs exhibited a maximum dose difference of 3% and 2% for PTV 1 mm and PTV 3 mm, respectively. The PTV 5 mm subvolume DVH showed a maximum dose difference of 1%.

### C. DVH of the breast

**Figure 6:**
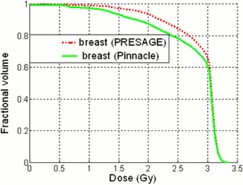
Dose‐volume histogram comparisons between the PRESAGE® and Pinnacle^3^ for breast.

Figure 6 illustrates the breast DVH comparison between Pinnacle^3^ and PRESAGE®. Deviations exist between 0.5 and 3 cGy. These discrepancies are likely due to edge artifacts, as the breast region of interest encompassed the entire PRESAGE®.

### D. Isodose line profiles

**Figure 7:**
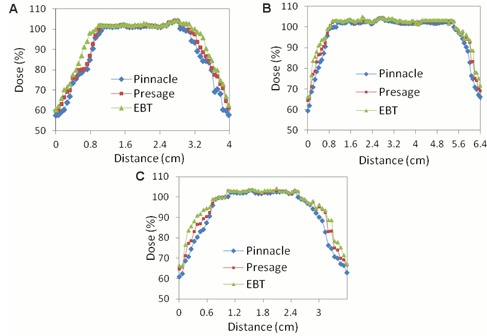
Line profiles of the Pinnacle^3^, PRESAGE®, and EBT2 film dose distributions from the axial slices (a)–(c) shown in Fig. 3.

Figure 7 illustrates line profile comparisons from the breast dosimeter between Pinnacle^3^, EBT2 film, and PRESAGE®. In general, the line plots show agreement among all three distributions, with a maximum difference of 5%. Some relatively minor differences can be recognized amongst the distributions. However, no systematic trends are apparent, and it is impossible to report whether the PRESAGE® or EBT2 distributions agree more with the Pinnacle^3^ distribution. The differences appear greatest at the periphery of the breast, as demonstrated by the three graphs shown in Fig. 7.

The line profile comparisons yielded a maximum difference amongst the three distributions of 2% within the central 80% of the field width. Another consideration is that the two measured distributions EBT2 and PRESAGE® actually correspond to two independent deliveries of the same treatment plan. Any variation in the mechanics of the delivery would also contribute to differences in the measured distribution.

### E. Gamma map comparisons

**Figure 8:**
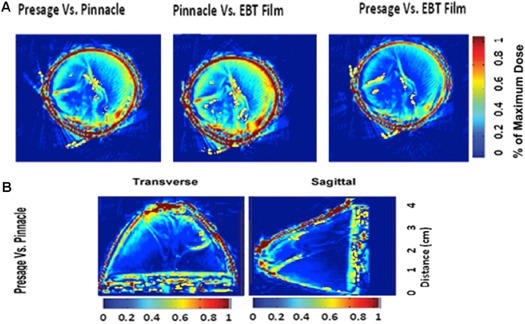
Gamma maps (a) (±3%/±3mm) between Pinnacle^3^, EBT2, and PRESAGE® for a region of PTV 5 mm on film plane B, as shown in Fig. 3; PRESAGE® and Pinnacle^3^ gamma distributions (b) (±3%/±3mm) in the transverse and sagittal planes for the PTV 5 mm intersecting film plane B, as shown in Fig. 3.

Figure 8(a) illustrates the gamma map comparisons (±3%/±3mm) amongst Pinnacle^3^, EBT2, and PRESAGE® for a region of PTV 5 mm on film plane B, as shown in Fig. 3.

The pass rates for the axial 2D gamma comparisons in Fig. 8(a) (±3%/±2mm) of EBT2 versus PRESAGE®, PRESAGE® versus Pinnacle^3^, and EBT2 versus Pinnacle3 were 88.4%, 90.6%, and 91.2%, respectively. The majority of failures in all three comparisons occur near the edge of the dosimeter in the outer 8 mm rind of the PRESAGE®. In this region, the PRESAGE® doses are likely inaccurate due to edge artifacts, and the Pinnacle^3^ dose may be inaccurate due to difficulty in modeling the buildup region. If this outer 8 mm rind is ignored, the pass rate rises to 95% for the 2D comparisons of PRESAGE® with Pinnacle^3^.

## V. CONCLUSIONS

An anthropomorphic breast PRESAGE® was created, and measurements of a clinically realistic IMRT treatment delivery were acquired and compared to the Pinnacle^3^ treatment planning system and GAFCHROMIC EBT2 film. The PRESAGE® and Pinnacle^3^ comparison for the PTV showed that 95.5% points passed the ±3%/±3mm criterion when the outer 8 mm of phantom data were discluded whereas EBT2 film agreement was 88.4% and 91.2% with PRESAGE® and Pinnacle^3^, respectively, for the film planes comparison (±3%/±3mm). Line profiles of the EBT2 film, Pinnacle^3^, and PRESAGE® were found to be within 2%, except for data within 8 mm of the edge of the dosimeter. The PTV DVH of PRESAGE® and Pinnacle^3^ showed a 5% dose difference at the edges. This work demonstrates the feasibility of fashioning PRESAGE® into an anthropomorphic shape for verification of breast IMRT, and it provides groundwork for future investigations into more complex anthropomorphic phantoms. The primary advantage of the PRESAGE®/optical CT system is the fact that it can produce true 3D dosimetry and this is highlighted in the dose‐volume histogram plots. But due to edge artifacts, high precision dose measurement is difficult at the edges. The losses of the signal at the edges of the dosimeter attenuate the laser light that produce the edge artifacts. To overcome these edge artifacts, optics and acquisition techniques should be improved to reduce the noise in reconstruction of the images.^(3)^ Moreover improvement in the optical CT scanner design and acquisition technique may lead to reduced edge artifacts.

## ACKNOWLEDGMENTS

This work was supported by the NIH Grant No. 5R01 CA 10083. We are also very thankful to HEC (Higher Education Commission of Pakistan) which provided the scholarship for the completion of this PhD research. We also acknowledge Matthew Pair, BS, for planning the IMRT breast case.

## References

[1] Oldham M , Sakhalkar H , Guo P , Adamovics J . An investigation of the accuracy of an IMRT dose distribution using two‐ and three‐dimensional dosimetry techniques. Med Phys. 2008;35(5):2072–80. 18561683 10.1118/1.2899995 PMC2562315

[2] Guo P , Adamovics J , Oldham M . Characterization of a new radiochromic three‐dimensional dosimeter. Med Phys. 2006;33(5):1338–45. 16752569 10.1118/1.2192888 PMC1616190

[3] Brady SL , Brown WE , Clift CG , Yoo S , Oldham M . Investigation into the feasibility of using PRESAGE®/optical‐CT dosimetry for the verification of gating treatments. Phys Med Biol. 2010;55(8):2187–201. 20348606 10.1088/0031-9155/55/8/005 PMC3018756

[4] Baldock C , De Deene Y , Doran S , et al. Polymer gel dosimetry. Phys Med Biol. 2010;55(5):R1–R63. 20150687 10.1088/0031-9155/55/5/R01 PMC3031873

[5] Doran SJ . The history and principles of chemical dosimetry for 3‐D radiation fields: gels, polymers and plastics. Appl Radiat Isot. 2009;67(3):393–98. 18675549 10.1016/j.apradiso.2008.06.026

[6] Brown S , Venning A , De Deene Y , Vial P , et al. Radiological properties of the PRESAGE and PAGAT polymer dosimeters. Appl Radiat Isot. 2008;66(12):1970–74. 18693028 10.1016/j.apradiso.2008.06.005

[7] Healy BJ , Gibbs A , Murry RL , Prunster JE , Nitschke KN . Output factor measurements for a kilovoltage X‐ray therapy unit. Australas Phys Eng Sci Med. 2005;28(2):115–21. 16060318 10.1007/BF03178702

[8] Hill R , Holloway L , Baldock C . A dosimetric evaluation of water equivalent phantoms for kilovoltage x‐ray beams. Phys Med Biol. 2005;50(21):N331–N344. 16237233 10.1088/0031-9155/50/21/N06

[9] Hill R , Kuncic Z , Baldock C . The water equivalence of solid phantoms for low energy photon beams. Med Phys. 2010;37(8):4355–63. 20879595 10.1118/1.3462558

[10] Hill R , Brown S , Baldock C . Evaluation of the water equivalence of solid phantoms using gamma ray transmission measurements. Radiat Meas. 2008;43(7):1258–64.

[11] Guo P , Adamovics J , Oldham, M . Characterization of a new radiochromic three‐dimensional dosimeter. Med Phys. 2006;33(5):1338–45. 16752569 10.1118/1.2192888 PMC1616190

[12] Seco J and Evans PM . Assessing the effect of electron density in photon dose calculations. Med Phys. 2006;33(2):540–52. 16532961 10.1118/1.2161407

[13] Sellakumar P , James Jebaseelan Samuel E , Supe SS . Water equivalence of polymer gel dosimeters. Radiat Phys Chem. 2007;76(7):1108–15.

[14] Adamovics J and Maryanski MJ . Characterization of PRESAGE: a new 3‐D radiochromic solid polymer dosimeter for ionising radiation. Radiat Prot Dosimetry. 2006;120(1‐4):107–12. 16782984 10.1093/rpd/nci555

[15] Guo P , Adamovics J , Oldham M . A practical three‐dimensional dosimetry system for radiation therapy. Med Phys. 2006;33(10):3962–72. 17089858 10.1118/1.2349686 PMC1780266

[16] Venning AJ , Nitschke KN , Keall PJ , Baldock C . Radiological properties of normoxic polymer gel dosimeters. Med Phys. 2005;32(4):1047–53. 15895589 10.1118/1.1881812

[17] Gorjiara T , Hill R , Kuncic Z , et al. Investigation of radiological properties and water equivalency of PRESAGE dosimeters. Med Phys. 2011;38(4):2265–74. 21626961 10.1118/1.3561509

[18] Sakhalkar H , Adamovics J , Ibbott G , Oldham M . A comprehensive evaluation of the PRESAGE/optical‐CT 3D dosimetry system. Med Phys. 2009;36(1):71–82. 19235375 10.1118/1.3005609 PMC2673667

[19] Sakhalkar H and Oldham M . Fast, high‐resolution 3D dosimetry utilizing a novel optical‐CT scanner incorporating tertiary telecentric collimation. Med Phys. 2008;35(1):101–11. 18293567 10.1118/1.2804616 PMC2504744

[20] Sakhalkar H , Adamovics J , Ibbott G , Oldham M . An investigation into the robustness of optical‐CT dosimetry of a radiochromic dosimeter compatible with the RPC head‐and‐neck phantom. J Phys Conf Ser. 2009;164(1):012059. 10.1088/1742-6596/164/1/012059 PMC2855979 20407594

[21] Sakhalkar H , Sterling HD , Adamovics J , Ibbott G , Oldham M . Investigation of the feasibility of relative 3D dosimetry in the Radiologic Physics Center Head and Neck IMRT phantom using presage/optical‐CT. Med Phys. 2009;36(7):3371–77. 19673232 10.1118/1.3148534 PMC2832043

[22] Newton J , Thomas A , Ibbott G , Oldham M . Preliminary commissioning investigations with the DMOS‐RPC optical‐CT Scanner. J Phys Conf Ser. 2010;250(1):012078. 10.1088/1742-6596/250/1/012078 PMC3015184 21218171

[23] Zhao L , Newton J , Oldham M , Das I , Cheng C , Adamovics J . Feasibility of PRESAGE for relative 3D dosimetry of small proton fields. Phys Med Biol. 2012;57(22):N431–N443. 23103526 10.1088/0031-9155/57/22/N431 PMC3844616

[24] Al‐Nowais S , Doran S , Kacperek A , Krstajic N , Adamovics J , Bradley D . A preliminary analysis of LET effects in the dosimetry of proton beams using PRESAGE and optical CT. Appl Radiat Isot. 2009;67(3):415–18. 18691895 10.1016/j.apradiso.2008.06.032

[25] Abdul Rahman A , Brauer‐Krisch E , Brochard T , et al. Sophisticated test objects for the quality assurance of optical computed tomography scanners. Phys Med Biol. 2011;56(14):4177–99. 21701054 10.1088/0031-9155/56/14/001

[26] Devic S , Seuntjens J , Sham E , et al. Precise radiochromic film dosimetry using a flat‐bed document scanner. Med Phys. 2005;32(7):2245–53. 10.1118/1.1929253 16121579

